# Association of Cytogenetics Aberrations and *IGHV* Mutations with Outcome in Chronic Lymphocytic Leukemia Patients in a Real-World Clinical Setting

**DOI:** 10.1055/s-0044-1779668

**Published:** 2024-02-12

**Authors:** Carolina Muñoz-Novas, Isabel González-Gascón-y-Marín, Iñigo Figueroa, Laura Sánchez-Paz, Claudia Pérez-Carretero, Miguel Quijada-Álamo, Ana-Eugenia Rodríguez-Vicente, María-Stefania Infante, María-Ángeles Foncillas, Elena Landete, Juan Churruca, Karen Marín, Victoria Ramos, Alejandro Sánchez Salto, José-Ángel Hernández-Rivas

**Affiliations:** 1 Servicio de Hematología, Hospital Universitario Infanta Leonor, Madrid, Spain; 2 Departamento de Medicina, Universidad Complutense, Madrid, Spain; 3 IBSAL, IBMCC, Centro de Investigación del Cáncer, Servicio de Hematología, Hospital Universitario de Salamanca, Universidad de Salamanca-CSIC, Salamanca, Spain

**Keywords:** chronic lymphocytic leukemia, *IGHV* mutation status, complex karyotype, overall survival, time to first treatment

## Abstract

Immunoglobulin heavy chain variable ( *IGHV* ) region mutations, *TP53* mutation, fluorescence in situ hybridization (FISH), and cytogenetic analysis are the most important prognostic biomarkers used in chronic lymphocytic leukemia (CLL) patients in our daily practice. In real-life environment, there are scarce studies that analyze the correlation of these factors with outcome, mainly referred to time to first treatment (TTFT) and overall survival (OS). This study aimed to typify *IGHV* mutation status, family usage, FISH aberrations, and complex karyotype (CK) and to analyze the prognostic impact in TTFT and OS in retrospective study of 375 CLL patients from a Spanish cohort. We found unmutated CLL (U-CLL) was associated with more aggressive disease, shorter TTFT (48 vs. 133 months, *p* < 0.0001), and shorter OS (112 vs. 246 months, *p* < 0.0001) than the mutated CLL. *IGHV3* was the most frequently used *IGHV* family (46%), followed by *IGHV1* (30%) and *IGHV4* (16%). *IGHV5-51* and *IGHV1-69* subfamilies were associated with poor prognosis, while *IGHV4* and *IGHV2* showed the best outcomes. The prevalence of CK was 15% and was significantly associated with U-CLL. In the multivariable analysis, *IGHV2* gene usage and del13q were associated with longer TTFT, while VH1-02, +12, del11q, del17p, and U-CLL with shorter TTFT. Moreover, VH1-69 usage, del11q, del17p, and U-CLL were significantly associated with shorter OS. A comprehensive analysis of genetic prognostic factors provides a more precise information on the outcome of CLL patients. In addition to FISH cytogenetic aberrations, *IGHV* and *TP53* mutations, *IGHV* gene families, and CK information could help clinicians in the decision-making process.

## Introduction

Chronic lymphocytic leukemia (CLL), the most frequent adult leukemia in the Western countries, shows a heterogeneous clinical course that reflects differences in disease biology. One-third of CLL patients has an indolent disease, with a life expectancy similar to that of age-matched healthy individuals; other patients have a benign phase of 3 to 10 years, after which the disease progresses, and approximately 15% of patients have an aggressive disease, with a dismal clinical outcome despite therapy. 1 2 Therefore, some patients require early treatment, while others only need a periodic follow-up. Multiple clinical and laboratory prognostic markers of CLL have been applied so far to try to predict the clinical course and outcome of this disease, highlighting Rai et al 3 and Binet et al 4 clinical staging systems, chromosomal abnormalities detected by fluorescence in situ hybridization (FISH), recurrent gene mutations, and immunoglobulin heavy chain variable ( *IGHV* ) locus gene mutation status. The complex karyotype (CK) as defined by ≥3 chromosomal abnormalities by conventional cytogenetics with stimulation techniques has emerged in the past years as an adverse prognostic and predictive marker not only to chemoimmunotherapy (CIT) treatments but also to novel agents. 5 6 Currently, the definition of CK in CLL is under discussion, since patients with five or more alterations do have a worse prognosis, which is not so evident in those who have three or four cytogenetic aberrations. 5

The *IGHV* mutation status is one of the most robust prognostic factors in CLL with a well-known ability to predict time to first treatment (TTFT), progression-free survival, and overall survival (OS). 7 8 Based on *IGHV* gene mutational status, CLL can be divided into mutated (M-CLL) and unmutated (U-CLL), with an arbitrary value of a 2% deviation from, or <98% identity with, the corresponding germline sequence. Though this classification is almost universal, some M-CLL cases were found to be more aggressive than expected, presenting a percentage of “borderline” mutations (97–97.9% *IGHV* identity) and, therefore, are intermediate between U-CLL and M-CLL. 9 M-CLL is associated with better clinical outcomes than U-CLL. This has been confirmed by numerous retrospective studies, observational studies from real life, clinical trials, and meta-analysis. 7 8 10 11 In addition, *IGHV* mutation status is one of the biomarkers included in the CLL-IPI, and current guidelines recommend its determination in every patient before treatment. However, unlike *TP53* mutation, *IGHV* mutation should only be performed once due to its immutability 12 13 14 ; therefore, *IGHV* mutation is important not only to establish prognosis but also for appropriate therapeutic decision-making in the age of new drugs; thus, most current guidelines include *IGHV* mutational status in treatment algorithms. The determination of this mutational state requires next-generation sequencing or reverse transcription polymerase chain reaction (PCR) techniques. New techniques are currently being explored in case molecular biology cannot be performed, such as multiparametric flow cytometry, with encouraging preliminary results. 15

Beyond mutation status, selective usage of individual *IGHV* genes has also been described in CLL, with a different distribution among gene rearrangements between different countries and an overuse of certain genes. For instance, *IGHV3* is the most frequent subgroup followed by *IGHV1* in Mediterranean countries, while *IGHV4* is more prevalent in China. Similarly, *IGHV1-69* is more frequently found in Mediterranean countries than Oriental countries. 16 17 18 Furthermore, specific used genes are associated with mutation status or even clinical outcome, such as *IGHV3-21* which harbors bad prognosis despite its association with mutation status 19 20 and published stereotyped subset #2, with poor results in both M-CLL and U-CLL. 21

Despite the meticulous characterization that has been made regarding to the *IGHV* families, due to the large number of used genes, a limited number of studies have focused on analyzing the prognosis they provide and their interaction with other prognostic factors, except for exceptional cases such as *IGHV3-21* . In this study, we retrospectively analyzed a large series of 375 unselected CLL patients, studying the relationship between *IGHV* gene usage and mutation status, FISH abnormalities, and conventional cytogenetics, including CK. We also assessed the prognostic impact of *IGHV* gene usage on TTFT and OS in our series, regardless of the treatment received.

## Materials and Methods

### Patients

We performed a retrospective multicenter analysis of a Spanish cohort of patients diagnosed with CLL from the electronic database of Cancer Research Center (Centro de Investigación del Cáncer—CIC), Salamanca, Spain. A total of 375 patients with comprehensive information about *IGHV* mutation status, family usage of *IGHV* and FISH analysis were included in this study. The laboratory data were exclusively collected at diagnosis. The diagnosis was based on the World Health Organization classification for CLL 22 and the International Workshop on Chronic Lymphocytic Leukemia (iwCLL) guidelines. 23 Clinical and biological variables include age, sex, Rai et al and Binet et al stages, lymphocytosis, somatic mutations of the *IGHV* gene, genetic abnormalities determined by FISH: deletions of 11q (del11q), 13q (del13q), 17p (del17p), trisomy 12 (+12), and karyotyping cytogenetic analysis. This study was performed in accordance with national and international guidelines (Declaration of Helsinki) and approved by the local ethics committees.

### *IGHV* Mutational Status

Analysis of the *IGHV* mutational status was performed locally at CIC laboratory, on peripheral blood CLL cell from fresh samples in tubes with ethylenediaminetetraacetic acid. *IGHV* gene rearrangements were amplified by reverse transcription-PCR in accordance with the European Research Initiative on CLL (ERIC) recommendations. 24 Mutation rates of ≥2% difference from germline were considered mutated, while unmutated disease had a <2% mutation rate.

### FISH

Clonal cytogenetic aberrations were studied by FISH analysis at CIC laboratory from peripheral blood samples obtained at diagnosis, using commercially available tests for detection (+12), and for del11q, del13q, and del17p (Vysis/Abbott Co., Downers Grove, Illinois, United States). Signal screening was carried out in at least 200 nucleated cells with well-delineated fluorescent spots. The sensitivity limit for the detection were >5 and >10% interphase cells with three signals and one signal, respectively, according to the cutoff from the laboratory.

### Cytogenetic Analysis

Cytogenetic analysis was also performed at CIC laboratory on peripheral blood samples. Cells were stimulated with CpG oligodeoxynucleotides and analyzed according to standard laboratory procedures. CK was defined by the presence of three or more chromosome abnormalities (numerical and/or structural) in the same clone, 5 25 and all types of alterations have been taken into account (unbalanced and balanced translocations, chromosomes addition, insertion, duplications, deletions, monosomies, or trisomies). We identified three subtypes of karyotypes: normal karyotype (NK), altered karyotype (AK): with one or two chromosomal abnormalities, and CK: at least three independent chromosomal abnormalities. CK cases with additional +12, +19, and +18 were not analyzed separately in the study. 26

### Statistical Analysis

The description of the quantitative values was made through the descriptive statistics of the median of the standard deviation and the 95% confidence interval. Fisher's exact test was used to detect statistically significant relationships between the categorical variables. To test statistically significant differences in continuous variables of scale, ratio, or interval, the Student's *t* test will be applied. Survival analysis was performed using Kaplan–Meier's curves for univariate analysis and Cox's regression for multivariate analysis. TTFT was calculated as the interval between diagnosis and the beginning of first-line treatment. OS was calculated from the time of diagnosis to death or to the last follow-up visit. Statistical analysis was performed using the program SAS v 9.4 and SPSS v 21.

## Results

### Patient Characteristics

A total of 375 patients were included in this study, 237 men (63%) and 138 women (37%). Median age at the time of diagnosis was 63 years (range, 25–89). Baseline characteristics for the cohort of CLL patients are summarized in Table 1 . Of all patients, 139 (37%) harbored a U-CLL and 236 (63%) had a M-CLL status. After a median follow-up time of 5.75 years (range 0–28), 70 patients had died (19%), while 172 (46%) required treatment.

**Table 1:** Baseline characteristics of patients

Variables		*N*	%
Age (y)	<65	194	52%
	≥65	181	48%
Sex	Male	237	63%
	Female	138	37%
Rai et al stage	0	226	60%
	I	75	22%
	II	29	8%
	III	7	2%
	IV	12	3%
	NR	26	7%
Binet et al stage	A	288	75%
	B	54	15%
	C	16	5%
	NR	17	5%
IGHV mutation status	Mutated	236	63%
	Unmutated	139	37%
Lymphocytes	<10,000	314	83.7%
	>10,000	55	14.7%
	NR	6	1.6%
FISH	No abnormality	141	38%
	13q	155	41%
	11q	16	4%
	17p	11	3%
	t12	52	14%
Karyotype	Normal	79	21%
	Altered	41	11%
	Complex karyotype	22	6%
	NP	233	62%

Abbreviations: FISH, fluorescence in situ hybridization; NP, not performed; NR, not reported.

### *IGHV* Family Usage and Relationship with Mutation Status

**Fig. 1:**
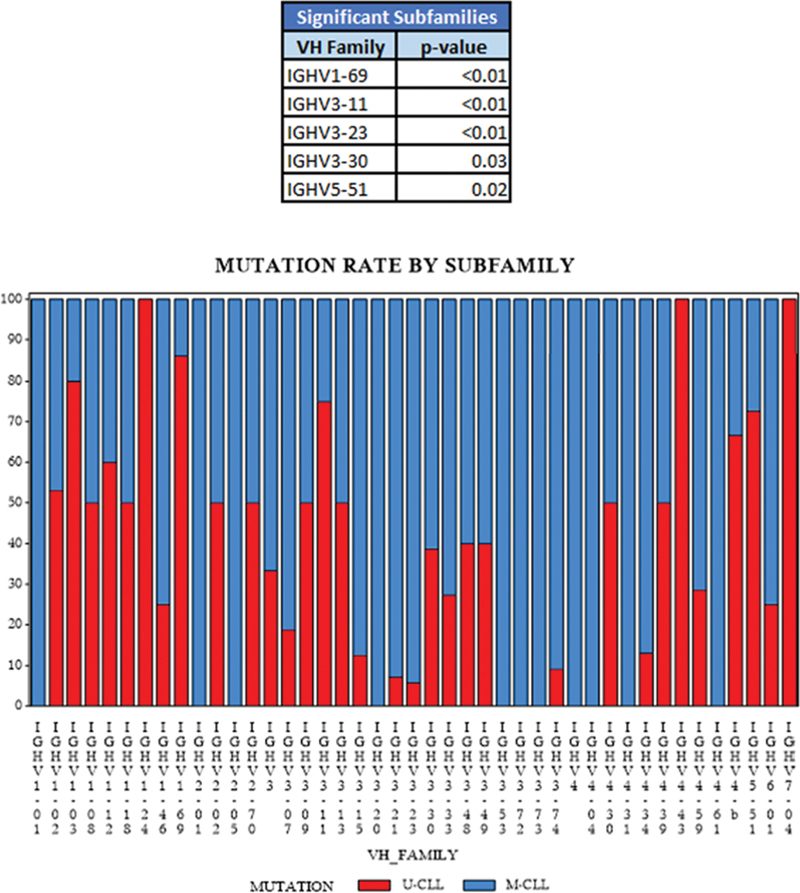
Relationship between ***IGHV*** **family usages and mutational status.** *IGHV* rearrangements that are significantly associated with mutation status are: *IGHV1-69* , *IGHV3-11* , *IGHV3-21* , *IGHV3-23* , and *IGHV5-51* . IGHV, immunoglobulin heavy chain variable; M-CLL, mutated chronic lymphocytic leukemia; U-CLL, unmutated chronic lymphocytic leukemia.

*IGHV3* was the most frequently used *IGHV* family (46%), followed by *IGHV1* (30%), *IGHV4* (16%), *IGHV2* (3%), and *IGHV5* (3%). *IGHV6* and *IGHV7* were detected in about 1% of the patients, respectively. Table 2 summarizes the proportion of patients who used each *IGHV* family and its relationship with mutation status, and Fig. 1 illustrates the proportion of each *IGHV* subfamily usage and its interaction with mutation status. *IGHV1* family had an excess of U-CLL (62%, *p* = 0.05) compared with the other *IGHV* families, probably favored by the contribution of the *IGHV1-69* rearrangement (25/29 U-CLL, *p* < 0.0001) ( Fig. 1 ). Conversely, *IGHV3* and *IGHV4* families were significantly associated with M-CLL mutational status. Within *IGHV3* , 127/166 had M-CLL ( *p* < 0.0001), indeed, most family usages from these families also had M-CLL, highlighting *IGHV3-21* (13/14 M-CLL, *p* = 0.021) and *IGHV3-23* (33/35, *p* < 0.0001). As an exception, *IGHV3-11* was significantly associated with U-CLL (5/6, *p* = 0.026). And within *IGHV4* (44/57 M-CLL, *p* = 0.016), the *IGHV4-34* subfamily was mostly related to M-CLL (20/23 M-CLL) and had a better TTFT than U-CLL ( *p* = 0.051). The *IGHV2* family had more cases with a M-CLL profile (67%), although the differences were not statistically significant. Notably, all cases of the *IGHV5* family belonged to the *IGHV5-51* subgroup, with a significant association with U-CLL (73%, *p* = 0.022).

**Table 2:** Family usage and mutational status of *IGHV* in the cohort of 375 CLL patients

*IGHV* family	*N* (%)	U-CLL, *n* (%)	M-CLL, *n* (%)	*p* -Value
Total *IGHV1*	111 (30)	69 (62.2)	42 (37.8)	0.000
Total *IGHV2*	12 (3)	4 (33)	8 (67)	NS
Total *IGHV3*	166 (45)	39 (23.5)	127 (76.5)	0.000
Total *IGHV4*	57 (16)	13 (22.8)	44 (77.2)	0.016
Total *IGHV5*	11 (3)	8 (73)	3 (27)	0.02
Other families	7 (1)	4 (57)	3 (43)	NS
Unknown	11 (2)	2 (18)	9 (82)	NS

Abbreviations: CLL, chronic lymphocytic leukemia; M-CLL, mutated chronic lymphocytic leukemia; U-CLL, unmutated chronic lymphocytic leukemia.

### Genomic Aberrations Detected by FISH, *IGHV* Mutation Status, and Family Usage

Next, we analyzed the incidence of cytogenetic aberrations detected by FISH (del11q, del13q, del17p, and +12) according to the *IGHV* mutation status ( Table 3 ). In our study, 100% of patients were carried out FISH and 62% of the patients harbored FISH alterations. As expected, del13q and normal FISH occurred more frequently in M-CLL patients ( *p* < 0.0001 in both cases). By contrast, del11q ( *p* = 0.0013), del17p ( *p* = 0.002), and +12 ( *p* = 0.0003) were associated with the U-CLL subgroup.

**Table 3:** Relationship between mutational status *IGHV* and genomic aberrations by FISH

FISH		*N*	IGHV		*p* -Value
			M-CLL HR	U-CLL	
del13q		155	116	39	0.0001
	TTFT		84 mo 0.291 (0.173–0.492)	50.5 mo	0.0001
	OS		149 mo 0.291 (0.123–0.691)	112 mo	0.005
Trisomy 12		52	21	31	0.0003
	TTFT		42 mo 0.879 (0.437–1.769)	38 mo	0.71
	OS		119 mo 0.992 (0.343–2.866)	115 mo	0.98
del11q		16	4	12	0.0013
	TTFT		10 mo 1.642 (0.472–5.707)	17 mo	0.43
	OS		87 mo 0.786 (0.080–7.764)	89 mo	0.84
del17p		11	1	10	0.0002
	TTFT		36 mo 0.713 (0.084–6.034)	37 mo	0.75
	OS		NC	81 mo	0.99
No abnormalities		141	94	47	0.0001
	TTFT		148 mo 0.224 (0.129–0.389)	51 mo	0.0001
	OS		286 mo 0.298 (0.121–0.735)	95 mo	0.0086

Abbreviations: FISH, fluorescence in situ hybridization; HR, hazard ratio; NC, not calculated; OS, overall survival; TTFT, time to first treatment.

Interestingly, *IGHV* mutation status had a significant impact on the outcomes among the different specific FISH subgroups ( Table 3 ). Patients with isolated del13q and M-CLL had longer TTFT and OS than patients with del13q and U-CLL ( Supplementary Fig. S1A, B ). Conversely, *IGHV* mutation status did not influence TTFT and OS of patients harboring +12 ( Supplementary Fig. S1C, D ). We did not find differences in TTFT and OS in patients with del11q and del17p ( Supplementary Fig. S1E–H ), but the number of cases was low in these cytogenetic alteration groups.

**Fig. 2:**
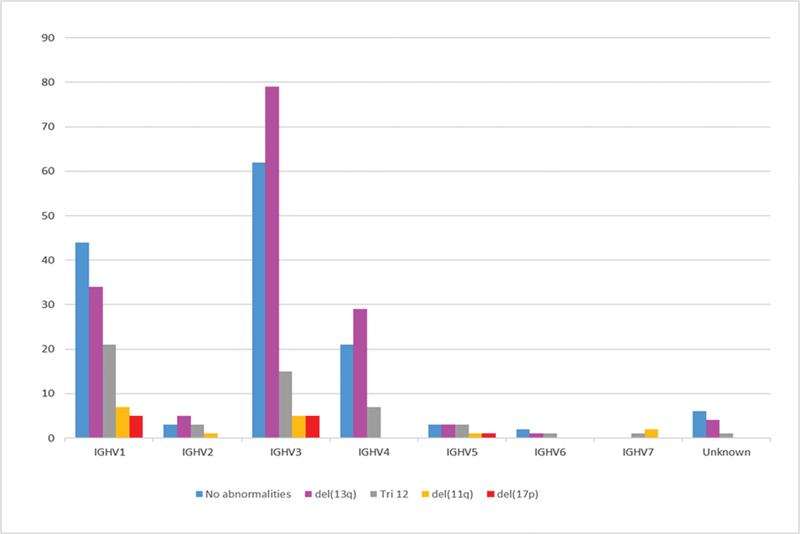
Relationship between ***IGHV*** **segments and FISH abnormalities.** FISH, fluorescence in situ hybridization; IGHV, immunoglobulin heavy chain variable.

We also describe the distribution of FISH abnormalities between each *IGHV* family and rearrangement ( Fig. 2 ). Interestingly, poor prognostic abnormalities were observed only in specific *IGHV* families and segments. For example, 46% (5/11) of the patients with del17p and 44% (7/16) of the patients with del11q belonged to the *IGHV1* family. On the other hand, the *IGHV4* family did not have cases with any of these two cytogenetic alterations. Moreover, the *IGHV2* , *IGHV4* , and the *IGHV3* families were enriched with cases belonging to the FISH-hierarchical good prognostic subgroups: 5/12 (42%) *IGHV2* , 79/166 (46%) *IGHV3* , and 29/57 (51%) *IGHV4* harbored del13q, respectively. In addition, FISH alterations with bad prognosis (del11q and del17p) were only represented in the *IGHV1* , *IGHV3* , *IGHV5* ( *IGHV5-51* subgroup), and *IGHV7* families.

### Relationship between Complex Karyotype, *IGHV* Mutation Status, and Family Usage

The relationship between CK, *IGHV* mutational status, and family usage was restricted to the 142 patients with karyotype information. NK was observed in majority of the cases (56%), followed by AK (29%) and CK in 22 patients (15%). A significant association between NK and M-CLL was detected in 61/79 patients (77%, *p* < 0.0001), while patients with CK had a significant association with U-CLL in 15/22 patients (68%, *p* = 0.001).

Moreover, within the subgroup of patients with CK, U-CLL conferred a shorter TTFT and more aggressive disease than for M-CLL ( *p* = 0.0195) ( Supplementary Fig. S2A, B ).

**Fig. 3 Distribution of:**
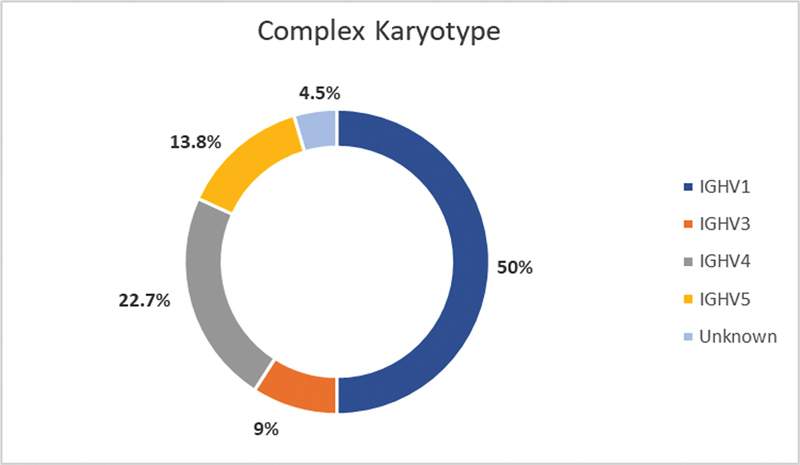
***IGHV*families between patients with CK (percentages of the total number of cases with complex karyotype).** CK, complex karyotype; IGHV, immunoglobulin heavy chain variable.

A biased usage of *IGHV* genes was detected in the CK subgroup, with a preference for *IGHV1* family (11/22 patients, 4 CK belonged to the family *IGHV1-69* and 4 to the *IGHV1-02* ), followed by *IGHV4* and *IGHV5* ( Fig. 3 ). None of the cases belonging to the *IGHV2* family had a CK.

### Outcome, *IGHV* Mutation, Family Usage, and Genomic Abnormalities

**Fig. 4 (A):**
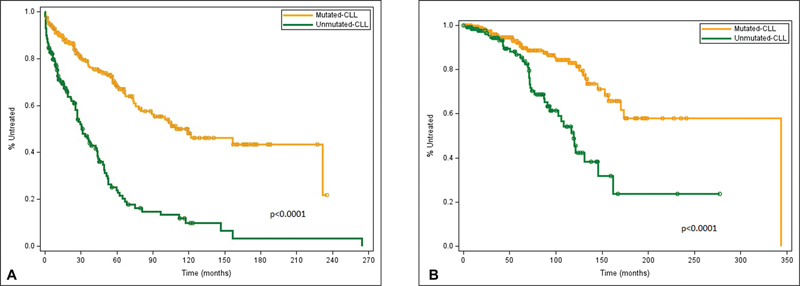
Time to first treatment in CLL patients with mutated and unmutated *IGHV.* **(B)** Overall survival in CLL patients with mutated and unmutated *IGHV* gene. CLL, chronic lymphocytic leukemia; IGHV, immunoglobulin heavy chain variable.

As expected, TTFT was significantly longer in patients with M-CLL compared with U-CLL (133 vs. 48 months, *p* < 0.0001) ( Fig. 4A ). Median OS was 246 months in the group of patients with M-CLL patients and 112 months in the U-CLL group ( *p* < 0.0001) ( Fig. 4B ).

In addition, we analyzed the impact of *IGHV* families, rearrangements, *IGHV* mutation status, FISH abnormalities, and CK on disease outcome. Due to the small size of some VH segment populations, we only included those with more than 10 cases. As emphasized in Supplementary Table S1 , in the univariate analysis, the variables significantly associated with shorter TTFT were *IGHV1* , VH1-02, VH1-69, VH5-51, +12, del11q, del17p, CK, and U-CLL. Conversely, *IGHV2* and del13q were significantly associated with a longer TTFT. Del11q, del17p, and U-CLL patients were related with shorter TTFT, as expected. In the multivariable analysis *IGHV2* , VH1-02, del11q, del17p, +12, and U-CLL were related with worse TTFT. Regarding OS, *IGHV-1* , *IGHV1-69* , del11q, del17p, +12, and U-CLL were also significantly associated with worse outcome, while *IGHV2* and del13q were associated with good prognosis in the univariate analysis. However, only VH1-69, del11q, del17p, and U-CLL were the variables associated with shorter OS in the multivariable analysis.

## Discussion

In this study of a large Spanish series of CLL patients with information about *IGHV* rearrangements ( *n* = 375), we analyzed the frequency and correlation of *IGHV* gene usage with other genetic variables, including FISH cytogenetic aberrations and CK, and clinical outcome. Previous studies have found a significant impact of *IGHV* mutation status on the prognosis of patients with CLL. 7 18 20 27 However, the relationships of *IGHV* gene usage with genomic aberrations by FISH and cytogenetic complexity as a biomarker at diagnosis are less frequent.

First of all, we confirmed the preferential use of *IGHV3* (46%) followed by *IGHV1* (30%), *IGHV4* (16%), *IGHV2* (3%), and *IGHV5* (3%). Our results are comparable with those observed in the populations of other Western countries which confirm usage of subfamilies with different geographic pattern among countries. 7 18 20 27 28 Within the *IGHV3* family, the most frequently found in our study, the distribution of the subfamilies in the study is similar to that of other published groups in the southern European region. 19 In our series, the *IGHV3* family was more associated with M-CLL as expected. The most frequent subfamily was *IGHV3-23* , most of them associated with M-CLL and showed a short TTFT than patients without this usage. Moreover, *IGHV3-21* is more common in Northern and Central Europe and Scandinavian CLL population, 28 29 30 and it is more infrequent in Southern European countries, 16 20 probably due to this reason we had a low frequency in our study (2.6%). In this family, we found a higher frequency of M-CLL cases, similar to previous reports. *IGHV3-21* family has been associated with an unfavorable prognosis independently of the *IGHV* mutational status, 28 29 30 31 32 but we could not confirm this result due to the small number of this subgroup in our cohort. As a novel finding, we identified *IGHV3-11* as a usage associated with dismal prognosis, with most of these patients belonging to the U-CLL subgroup and showing a shorter TTFT and OS than patients without this usage, is in line with previous work from our group. 16 33 The significance of these results could not be proved and should be taken cautiously due to the low representation of this subfamily in our study.

*IGHV1* usage, regardless of mutational status, was associated with a worse prognosis and worse results than the rest of the families, with majority of U-CLL cases. The most frequently found subfamily was *IGHV1-69* , similar to other studies carried out in countries of the Western environment. 34 As described in other series, we confirmed that *IGHV1-69* distinguishes a uniformed group of patients with adverse outcome. 35 In our study, we observed a significant relationship with U-CLL and a lower OS than patients without this family, and the multivariable analysis showed a strong association with worse survival ( Supplementary Table S1 ).

With respect to the *IGHV4* family, the patients more frequently had a mutated pattern. Globally, this group presented with a long TTFT compared with the rest of the patients, especially in the *IGHV4-34* subfamily, the most common in our study and in other similar ones. 36 Interestingly, in patients with *IGHV4* , we did not find poor prognosis FISH alterations (del11q and del17p), as previous reports 37 and conversely, del13q alone was observed in half of the patients. Our study further expands the evidence suggesting that this subset represents a group of patients with indolent disease.

In relation to the families found less frequently in our study, in the family *IGHV2* , 67% of cases were associated with M-CLL, with differences in TTFT and OS in univariable analysis, but not in multivariable analysis, probably due to low representation of *IGHV2* family. In our study, *IGHV2* showed absence of CK, low percentage of bad prognosis mutations (only one case with del11q and no cases with del17p), which could suggest a good prognosis we found in this subgroup.

All cases of *IGHV5* family belonged to *IGHV5-51* usage. Previous studies suggest that this family should be studied to clarify the inferior prognosis in these patients. 16 38 In our study, we found a significant association between *IGHV5-51* and U-CLL, and all patients except one were female. It is remarkable the dismal outcome in this subgroup in univariate analysis is the family with the shortest TTFT (hazard ratio 3.08, *p* = 0.01). Despite the poor prognosis of this subfamily, only 2 out of the 11 patients had high-risk cytogenetic abnormalities.

Finally, similar to other published series, the low representation of the *IGHV6* and *IGHV7* families does not allow the estimation of better or worse clinical course.

Summarizing, our results point out that belonging to the *IGHV2* family could be a good prognostic factor, while the *IGHV1* family and some of their specific usages, mainly VH1-69 and VH1-02, might be associated with a dismal outcome.

We also analyzed the cytogenetic abnormalities detected by FISH and karyotyping, and the relations with mutation status of *IGHV* . A German university study used FISH analysis to demonstrate that about 80% of CLL patients had a least one genomic alteration in all diagnoses, and it was established that patients with a sole del13q and +12 had a better OS than patients with del17p or del11q. In our study, we showed the poor prognosis that U-CLL confers on the patients with isolated del13q, with a shorter TTFT and OS, similar to previous reports. 39 40 41 In the group of patients with +12 as the only cytogenetic aberration, patients with U-CLL or M-CLL did not show any significant differences in TTFT and OS between both groups based on the *IGHV* mutational status, as previously reported. 39 Regarding the poor cytogenetic risk (del11q or del17p) and *IGHV* mutational status, we did not find differences, probably due to the low representation.

Recent studies have shown that current FISH analysis, according to Dohner's hierarchical model, underestimates the true genetic complexity revealed by chromosome banding analysis. 42 In fact, 22 to 36% of CLL cases with “normal” FISH carry chromosomal aberration at karyotype. In particular, CK, defined by the presence of at least three chromosome lesions in the same clone, can be detected in 14 to 34% of CLL cases and is emerging as a new negative prognostic biomarker associated with an adverse outcome and worse response to CIT as well as to novel agents. 6 As in these studies, we also analyzed the CK. In our study, CK cases were relatively rare, representing 15% of the patients, according to other published studies 6 26 43 44 with a significantly higher proportion of U-CLL (68%). In addition, we observed that the combination allows to identify patients with M-CLL who are characterized by a more indolent disease and with TTFT longer than U-CLL, similar to the results obtained by the Italian group. 45 The results found in this work of the correlations of the *IGHV* mutational status, the cytogenetic alterations by FISH and the CK reflect the need for additional clinical studies with a larger number of patients, generally in the context of randomized clinical trials.

It is important to consider that the guidelines from the iwCLL recommend testing for *IGHV* gene mutation status at baseline in all patients diagnosed with CLL. 46 47 In addition, FISH analysis should be performed before any line of treatment of CLL patients. 47 Moreover, karyotyping could be introduced in the next future as a recommended test before the onset of therapy in CLL. In fact, FISH, karyotyping, and *IGHV* mutational status are probably the most powerful and validated clinical prognostic biomarker used in our daily practice. 41 42 48

This study has several limitations: the retrospective nature of the study and the impact of an inherent referral bias on our results. Even though we would have to analyze our results based on the origin of the patients, it was not feasible due to the size of our series and the scarce information about the individual ethnic origin of the patients thought the vast majority of patients were of Caucasian origin. In some occasions, the number of cases and the relatively small sample size of some groups did not reach the level required to perform statistically significant analysis. Among other additional limitations are those related to missing information about stereotypes of *IGHV* and its absence in this analysis of main mutations of genes related with CLL. Finally, the patients of this study were treated almost exclusively with CIT (93%) and this could be biasing the data about survival. Current recommended treatment is not CIT but molecularly targeted drugs. No *TP53* mutation data are available.

## Conclusion

In conclusion, the interactions between *IGHV* gene usage, mutation status, FISH, and CK may help provide more precise information about the prognosis of patients diagnosed with CLL and its clinical course. Further real-world studies similar to those described here are needed in the context of treatment with new oral small molecules and new anti-CD20 monoclonal antibodies.
