# Supplementary material for: Association of Cytogenetics Aberrations and IGHV Mutations with Outcome in Chronic Lymphocytic Leukemia Patients in a Real-World Clinical Setting
Source: Glob Med Genet. 2024 Feb 12;11(1):59–68. doi: 10.1055/s-0044-1779668 (PMC10861322; doi:10.1055/s-0044-1779668)
Supplement: Supplementary file 1 — Supplementary Material [file 10-1055-s-0044-1779668-s2300096.pdf]

Supplementary Material

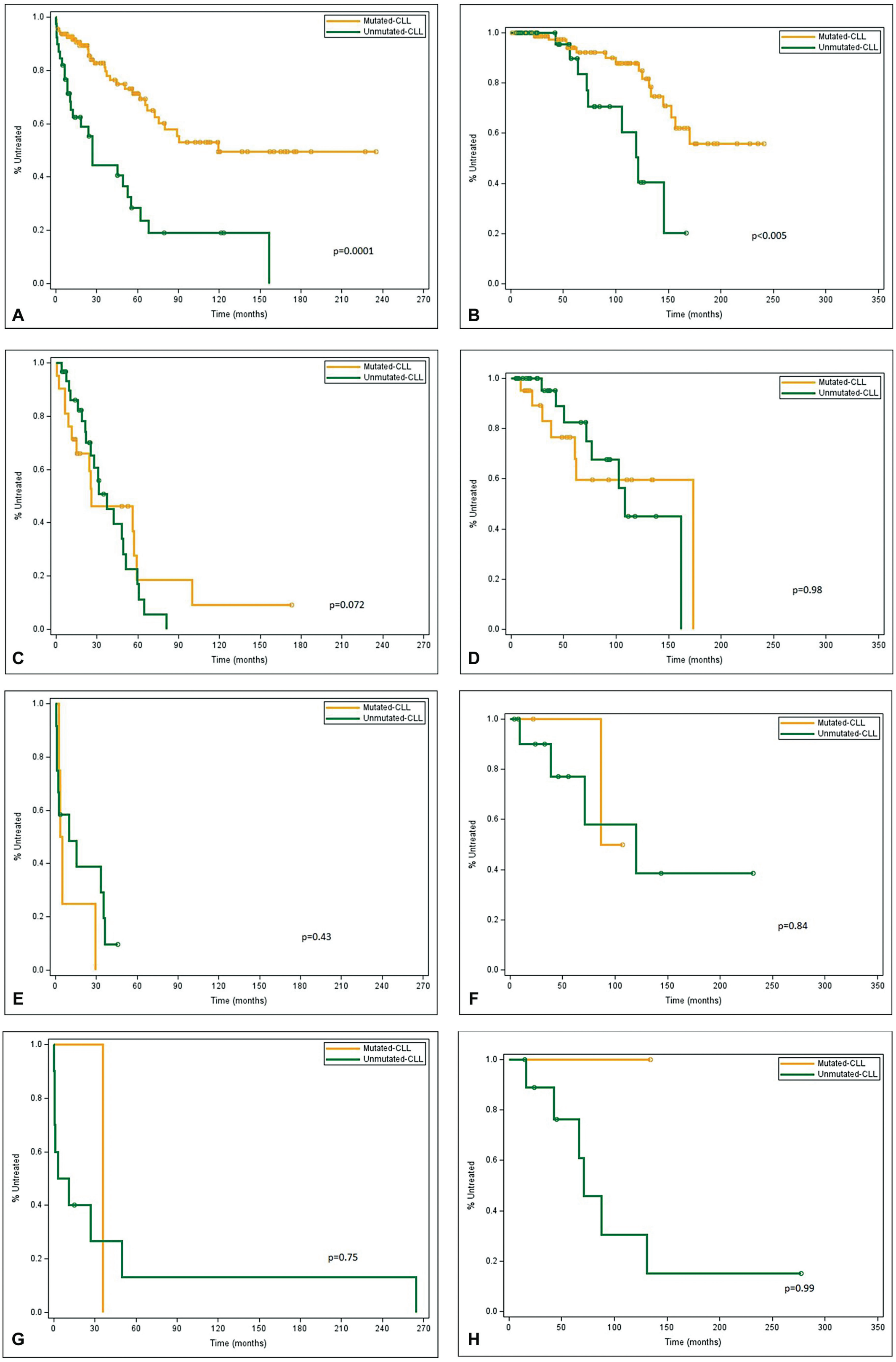

Supplementary Fig. S1 (A) Time to first treatment of patients with isolated del 13q based on *IGHV* mutational status. (B) Overall survival of patients with isolated del 13q based on *IGHV* mutational status. (C) Time to first treatment of patients of trisomy 12 based on *IGHV* mutational status. (D) Overall survival of patients with trisomy 12 based on *IGHV* mutational status. (E) Time to first treatment of patients with del 11q based on *IGHV* mutational status. (F) Overall survival of patients with del 11q based on *IGHV* mutational status. (G) Time to first treatment of patients with del 17p based on *IGHV* mutational status. CLL, chronic lymphocytic leukemia; *IGHV*, immunoglobulin heavy chain variable.

 © 2024. The Author(s).

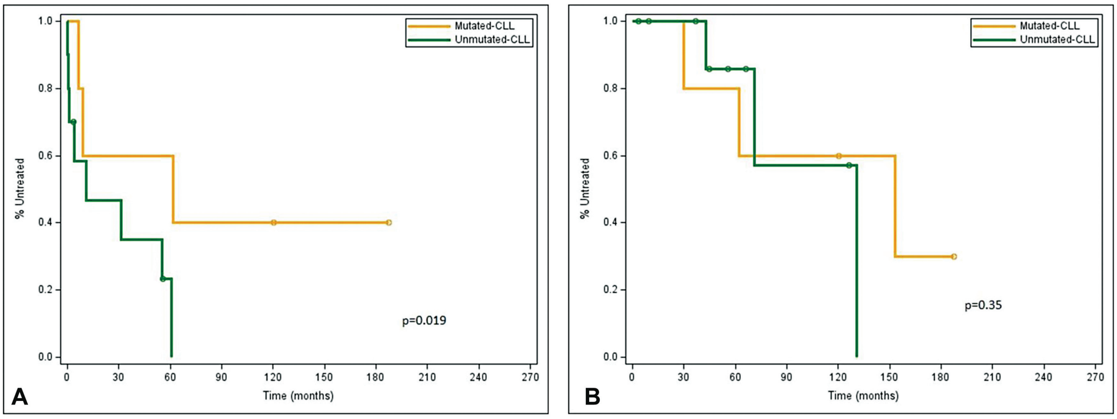

Supplementary Fig. S2 Complex karyotype. (A) Time to first treatment of patients with complex karyotype based on *IGHV* mutational status. (B) Overall survival of patients with complex karyotype based on *IGHV* mutational status. CLL, chronic lymphocytic leukemia.

 © 2024. The Author(s).

Supplementary Table S1 Univariate and multivariate analyses for TTFT and OS

	Univariate analysis (TTFT)			Multivariate analysis (TTFT)			Univariate analysis (OS)			Multivariate analysis (OS)		
	HR	95% CI	*p*-Value	HR	95% CI	*p*-Value	HR	95% CI	*p*-Value	HR	95% CI	*p*-Value
IGHV-1	1.60	1.23–2.08	<0.01	1.11	0.78–1.54	0.54	1.64	1.12–2.39	<0.01	1.09	0.71–1.68	0.68
IGHV-2	0.39	0.23–0.68	<0.01	0.44	0.24–0.78	<0.01	0.43	0.19–0.99	0.04	0.45	0.19–1.09	0.07
IGHV-3	0.82	0.60–1.10	0.19				0.89	0.58–1.37	0.60			
IGHV-4	0.73	0.46–1.156	0.18				0.55	0.27–1.13	0.10			
IGHV-5	3.08	1.57–6.02	<0.01				2.72	1.00–7.42	0.05			
VH1	1.04	0.78–1.34	0.77				1.12	0.78–1.67	0.56			
VH1-02	1.58	1.32–1.89	<0.01	1.53	1.23–1.88	<0.01	1.08	0.80–1.46	0.60			
VH1-08	0.62	0.34–1.01	0.05				0.93	0.66–1.32	0.71			
VH1-69	1.66	0.94–2.90	0.08	1.12	0.60–2.06	0.72	3.12	1.62–6.00	<0.01	2.14	1.05–4.351	0.03
VH2	0.95	0.91–1.01	0.05				0.95	0.88–1.03	0.19			
VH3-07	0.97	0.94–1.05	0.97				1.03	0.97–1.11	0.28			
VH3-11	1.92	0.61–6.02	0.26				2.28	0.31–16.47	0.42			
VH3-21	0.82	0.30–2.20	0.69				0.86	0.21–3.50	0.84			
VH3-23	0.47	0.21–1.06	0.68				1.04	0.42–2.47	0.92			
VH3-30	0.99	0.93–1.04	0.53				0.99	0.96–1.03	0.64			
VH3-33	0.98	0.97–1.02	0.76				0.99	0.91–1.07	0.74			
VH3-74	1.57	0.58–4.22	0.38				2.41	0.76–7.62	0.13			
VH4-34	0.68	0.32–1.45	0.31				0.53	0.17–1.68	0.28			
VH5-51	3.08	1.57–6.02	<0.01	1.08	0.52–2.23	0.83	2.72	1.00–7.42	0.05			
Del 11q	7.37	4.61–12.95	<0.01	6.01	3.43–10.78	<0.01	2.91	1.35–6.29	<0.01	2.64	1.16–5.97	0.02
Del 17p	2.82	1.49–5.32	<0.01	2.20	1.06–4.53	0.03	3.26	1.58–6.72	<0.01	2.70	1.23–5.92	0.01
Tri 12	2.03	1.48–2.79	<0.01	1.48	1.02–2.16	0.03	1.76	1.12–2.78	0.01	1.37	0.81–2.32	0.23
Del 13q	0.73	0.53–0.97	0.03	0.78	0.55–1.07	0.12	0.56	0.36–0.86	<0.01	0.88	0.53–1.45	0.61
CK	1.85	1.12–3.03	0.01	1.52	0.91–2.52	0.11	1.68	0.87–3.23	0.12			
U-CLL	3.70	2.82–4.84	<0.01	3.19	2.35–4.37	<0.01	3.47	2.36–5.11	<0.01	2.68	1.71–4.20	<0.01

Abbreviations: CI, confidence interval; CK, complex karyotype; HR, hazard ratio; M-CLL, mutated chronic lymphocytic leukemia; OS, overall survival; TTFT, time to first treatment; U-CLL, unmutated chronic lymphocytic leukemia.

 © 2024. The Author(s).
